# Supplementary material for: Unraveling Seasonal Allocation of Soluble Sugars, Starch and Proline in Sternbergia lutea
Source: Plants (Basel). 2023 Aug 24;12(17):3043. doi: 10.3390/plants12173043 (PMC10490230; doi:10.3390/plants12173043)
Supplement: Supplementary file 1 [file plants-12-03043-s001.zip › plants-2553215-supplementary.pdf]

Supplementary Figures

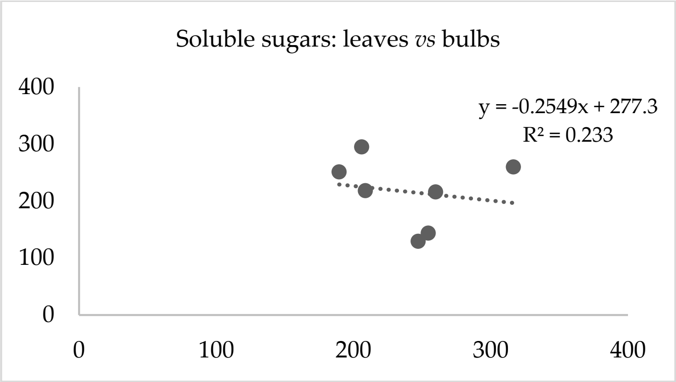

Figure S1. Soluble sugar content of leaves (mg g^{-1}) (vertical axis) plotted *versus* soluble sugars of bulbs (mg g^{-1}) (horizontal axis); the black circles correspond to mean values detected during the leafy stage of *S. lutea,* from October to April.

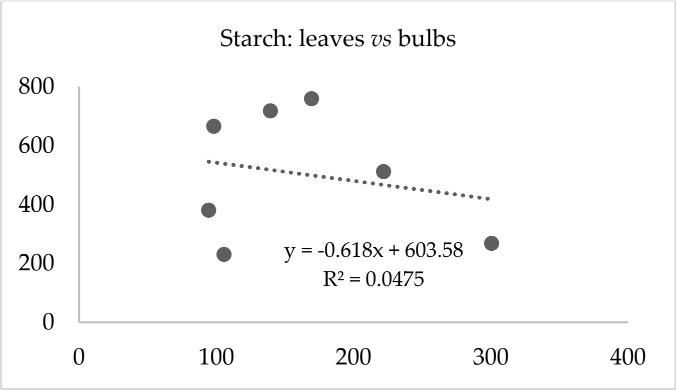

Figure S2. Starch of leaves (mg g^{-1}) (horizontal axis) plotted *versus* starch of bulbs (mg g^{-1}) (vertical axis); the black circles correspond to mean values detected during the leafy stage of *S. lutea,* from October to April.

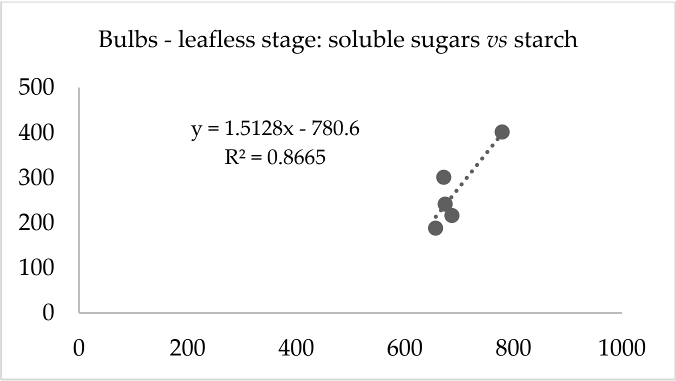

Figure S3a. Starch of bulbs (mg g^{-1}) (horizontal axis) plotted *versus* soluble sugars of bulbs (mg g^{-1}) (vertical axis); the black circles correspond to mean values detected in the bulbs during the leafless stage of *S. lutea*, from May to September.

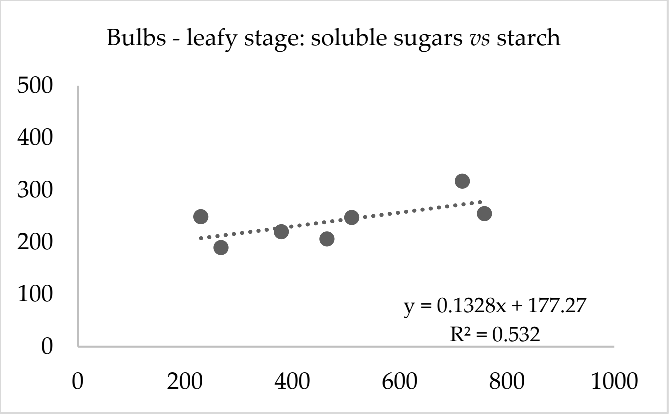

Figure S3b. Starch of bulbs (mg g^{-1}) (horizontal axis) plotted *versus* soluble sugars of bulbs (mg g^{-1}) (vertical axis); the black circles correspond to mean values detected during the leafy stage in the bulbs of *S. lutea*, from October to April.

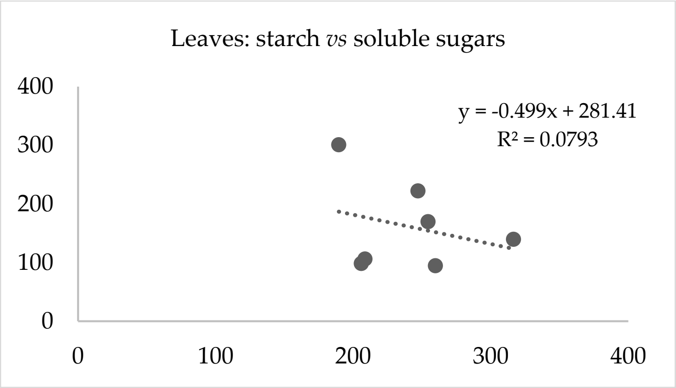

Figure S4. Soluble sugars of leaves (mg g^{-1}) (horizontal axis) plotted *versus* starch of leaves (mg g^{-1}) (vertical axis); the black circles correspond to mean values detected during the leafy stage of *S. lutea* from October to April.

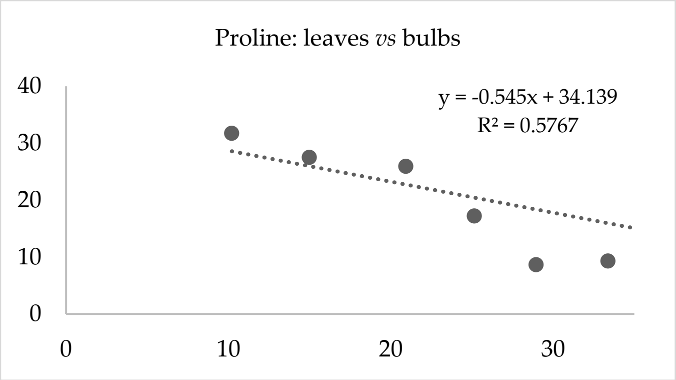

Figure S5. Free proline accumulation in leaves (μg g^{-1}) (vertical axis) plotted *versus* free proline accumulation in bulbs (horizontal axis); the black circles correspond to mean values detected during the leafy stage of *S. lutea,* from October to April.

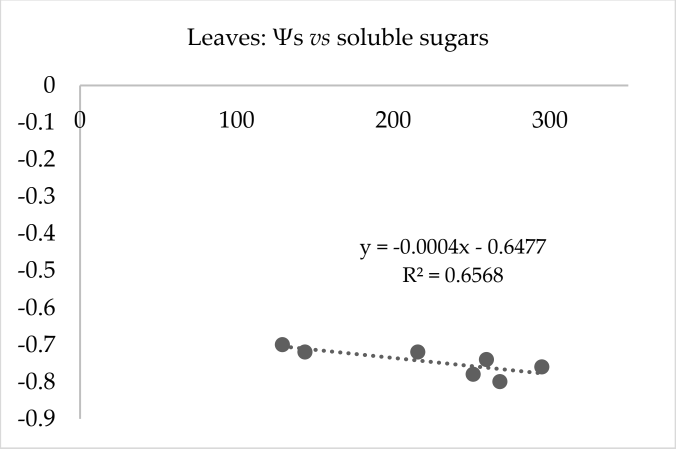

Figure S6. Soluble sugars of leaves (mg g^{-1}) (horizontal axis) plotted *versus* leaf osmotic potential, Ψs (MPa) (vertical axis); the black circles correspond to mean values detected during the leafy stage of *S. lutea,* from October to April.

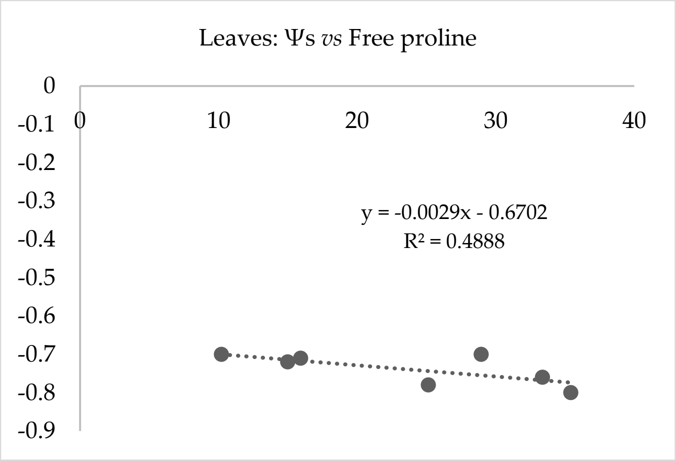

Figure S7. Free proline accumulation in leaves (µg g^{-1}) (horizontal axis) plotted *versus* leaf osmotic potential, Ψs (MPa) (vertical axis); the black circles correspond to mean values detected during the leafy stage of *S. lutea,* from October to April.

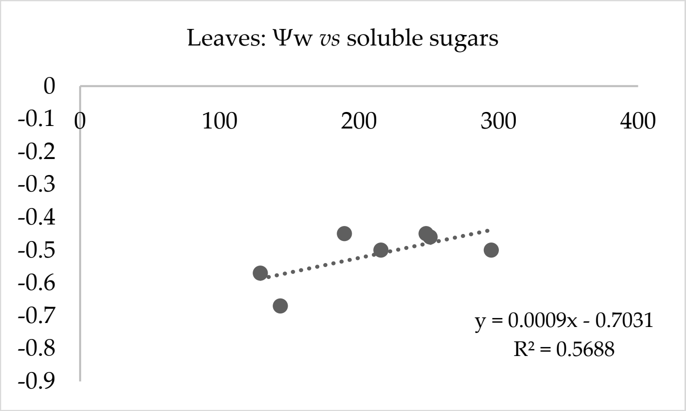

Figure S8. Soluble sugars of leaves (mg g^{-1}) (horizontal axis) plotted *versus* leaf water potential, Ψw (MPa) (vertical axis); the black circles correspond to mean values detected during the leafy stage of *S. lutea,* from October to April.

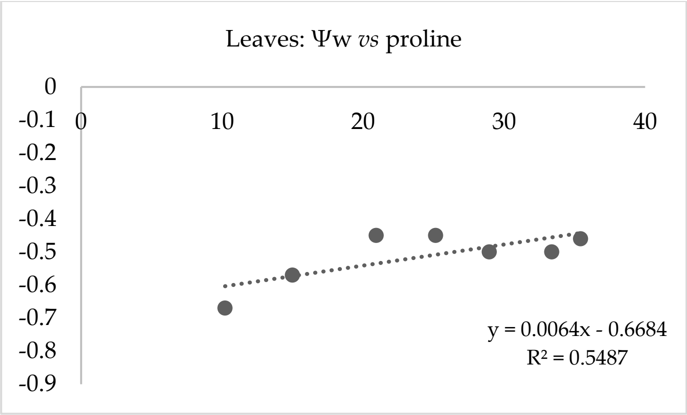

Figure S9. Free proline accumulation in leaves (μg g^{-1}) (horizontal axis) plotted *versus* leaf water potential, Ψw (MPa) (vertical axis); the black circles correspond to mean values detected during the leafy stage of *S. lutea* from October to April.
